# Anti-Inflammatory Effects of a Novel Acetonitrile–Water Extract of Lens Culinaris against LPS-Induced Damage in Caco-2 Cells

**DOI:** 10.3390/ijms25073802

**Published:** 2024-03-28

**Authors:** Fatima Maqoud, Antonella Orlando, Domenico Tricarico, Marina Antonacci, Annamaria Di Turi, Gianluigi Giannelli, Francesco Russo

**Affiliations:** 1 Functional Gastrointestinal Disorders Research Group, National Institute of Gastroenterology IRCCS “Saverio de Bellis”, 70013 Castellana Grotte, BA, Italy; fatima.maqoud@irccsdebellis.it (F.M.); antonella.orlando@irccsdebellis.it (A.O.); 2 Section of Pharmacology, Department of Pharmacy-Pharmaceutical Sciences, University of Bari ‘Aldo Moro’, 70121 Bari, BA, Italy; domenico.tricarico@uniba.it (D.T.); marina.antonacci@uniba.it (M.A.); annamaria.dituri@uniba.it (A.D.T.); 3 Scientific Direction, National Institute of Gastroenterology IRCCS “Saverio de Bellis”, 70013 Castellana Grotte, BA, Italy; gianluigi.giannelli@irccsdebellis.it

**Keywords:** ion channels, functional gastrointestinal disorders, transporters, transient receptor potential, aquaporin, ATP-sensitive K^+^ channels, calcium-activated K^+^ channels, voltage-dependent sodium channels, irritable bowel syndrome (IBS), intestinal dismicrobism

## Abstract

Natural compounds like flavonoids preserve intestinal mucosal integrity through their antioxidant, anti-inflammatory, and antimicrobial properties. Additionally, some flavonoids show prebiotic abilities, promoting the growth and activity of beneficial gut bacteria. This study investigates the protective impact of Lens culinaris extract (LE), which is abundant in flavonoids, on intestinal mucosal integrity during LPS-induced inflammation. Using Caco-2 cells as a model for the intestinal barrier, the study found that LE did not affect cell viability but played a cytoprotective role in the presence of LPS. LE improved transepithelial electrical resistance (TEER) and tight junction (TJ) protein levels, which are crucial for barrier integrity. It also countered the upregulation of pro-inflammatory genes TRPA1 and TRPV1 induced by LPS and reduced pro-inflammatory markers like TNF-α, NF-κB, IL-1β, and IL-8. Moreover, LE reversed the LPS-induced upregulation of AQP8 and TLR-4 expression. These findings emphasize the potential of natural compounds like LE to regulate the intestinal barrier and reduce inflammation’s harmful effects on intestinal cells. More research is required to understand their mechanisms and explore therapeutic applications, especially for gastrointestinal inflammatory conditions.

## 1. Introduction

The intestinal lining serves as a critical protective barrier, effectively preventing the absorption of pathogens, toxins, and allergens into the bloodstream. This safeguarding mechanism relies on intricate transcellular and paracellular pathways essential for water and ion absorption, thereby maintaining the delicate equilibrium of the gut. However, when the integrity of this barrier weakens, it initiates a cascade of immune responses, leading to inflammation in the gut tissue and a comprehensive disruption of the barrier’s functionality [1].

Key components in this process are the tight junctions (TJs) located on the apical region of the epithelial cells’ lateral membrane. These TJs, composed of claudin, occludin, JAM-A, and intracellular plaque proteins like zonula occludens (ZOs), establish vital connections between adjacent cells. Collectively, they form a selective barrier that meticulously regulates the movement of molecules and ions, ensuring the integrity of the tissue [2]. Additionally, the mucous gel layer, immunoglobulin A, and antibacterial peptides significantly contribute to fortifying the strength and functionality of the gut barrier, thereby safeguarding overall gut health [3].

Fundamental in gut homeostasis is the role of aquaporins (AQPs), where AQP1, AQP2, AQP3, and AQP8 actively participate in regulating the transport of water and solutes through gut epithelia. These processes are essential for osmoregulation and digestive and absorptive functions. Specifically, AQP8 directly influences colon water balance, and any alterations in its expression can manifest in symptoms such as diarrhea or constipation, impacting the consistency and frequency of bowel movements [4,5].

This delicate balance can be compromised by several external and internal factors [6]. External inflammatory triggers, such as infections, toxins, or allergens, have the potential to activate an inflammatory response in the gut lining, leading to an increase in epithelial barrier permeability. Internally, factors like cytokines, lipopolysaccharide (LPS), and immune response signals can affect TJ protein expression, with certain cytokines, such as tumor necrosis factor-alpha (TNF-α), causing harm to TJ protein expression and function, thus further compromising the overall integrity of the epithelial barrier [7].

Notably, LPS, acting through its TLR-4 receptor, has the capability to activate members of the TRP superfamily, including TRPV1 and TRPA1, both known for their involvement in inflammation [8]. Activation of these channels by various factors, including those produced by the intestinal microbiota, can elicit pro-inflammatory and anti-inflammatory responses. Understanding the contributions of TLR-4, TRPV1, and TRPA1 to LPS-induced inflammation is important for the development of targeted therapeutic strategies [9]. Studies indicate that the activation of TRPV1 and TRPA1 can influence TJ integrity, potentially increasing permeability in various tissues, including the gastrointestinal tract [10].

Flavonoids, which are natural compounds abundant in fruits, vegetables, and plants, have been shown to actively contribute to the maintenance of intestinal barrier integrity. Notably, extensively studied flavonoids like quercetin and naringenin have demonstrated their potential to enhance the function of the intestinal barrier [11,12], upregulating the levels of TJ proteins in the intestinal epithelium. Beyond this, their positive impact on intestinal barrier function may extend to the regulation of inflammatory responses, effectively preventing the infiltration of pro-inflammatory molecules and thereby reducing the risk of chronic inflammation in the gut [13,14].

Among flavonoids, the Lens culinaris Medik extract (LE), prepared according to the method proposed by Di Turi et al. [15], stands out for its notable health benefits and bioactive properties; its liquid chromatography-mass spectrometry (LC-MS) analysis revealed a high content of flavonoids. LE at a concentration range of 0.1–5 mg/mL in in vitro studies has provided evidence of its potential to protect against cytotoxic damage induced by cisplatin, irinotecan, and doxorubicin on different cell lines [15,16]. The anti-inflammatory potential of LE positions it as a promising candidate for managing inflammatory conditions.

In this framework, our primary aim was to comprehensively explore the effects of LE on intestinal barrier function and its influence on the ion channels, such as TRPV1, TRPA1 and AQP, associated with pain relief and anti-inflammatory effects. Utilizing a Caco-2 cell monolayer model treated with LPS alone or in combination with LE, we employed different methods, including TEER measurement, viability assays, Western blotting analysis, and qRT-PCR analysis. Additionally, we investigated the effects of LPS and LE on TRPV1 currents in Caco-2 cells through whole-cell recording using the patch-clamp technique under physiological conditions.

## 2. Results

### 2.1. Effects of LE on the Viability of Caco-2 Cells under Different LPS Treatments

**Figure 1:**
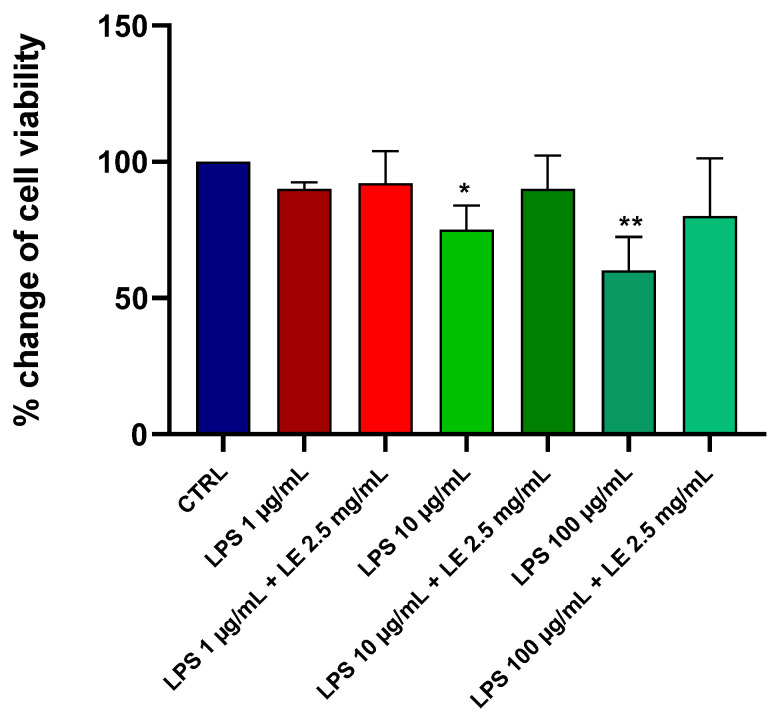
Effects of different concentrations of LPS, alone or in combination with LE, on the viability of Caco-2 cells after 24 h of treatment. The viability of the cells was measured using the MTT assay. The results are expressed as the mean ± SD of five independent experiments with six replicates per experimental condition in each experiment. * *p* < 0.05, ** *p* < 0.01 vs. the control group (CTRL).

The impact of LPS at varying concentrations on the viability of Caco-2 cells was evaluated. Following a 24 h treatment period, LPS induced a dose-dependent reduction in cell viability, as evaluated by the MTT assay (Figure 1). Notably, concentrations of 100 and 10 μg/mL of LPS led to significant reductions of approximately 40% and 25%, respectively. In contrast, incubation with a 1 μg/mL concentration resulted in a minor, albeit statistically non-significant, reduction of around 10% compared to that of the control (CTRL).

The viability of Caco-2 cells subjected to LE concentrations ranging from 1 to 3 mg/mL for either 24 or 48 h remained unaltered when compared to the control group (*p* > 0.05). These data align with safety ranges documented in other studies [15,16], where LE has demonstrated safety within the concentration range from 1 to 3 mg/mL across various cell types. Co-incubation of LPS at concentrations of 100, 10, and 1 μg/mL with LE (2.5 mg/mL) exhibited a cytoprotective effect against LPS-induced damage in Caco-2 cells (Figure 1). These findings suggest that the utilized concentration range of the liposomal extract had no discernible toxic effects on the viability of the tested cells.

### 2.2. Preliminary Data and Choice of Concentration of LE and LPS

**Figure 2:**
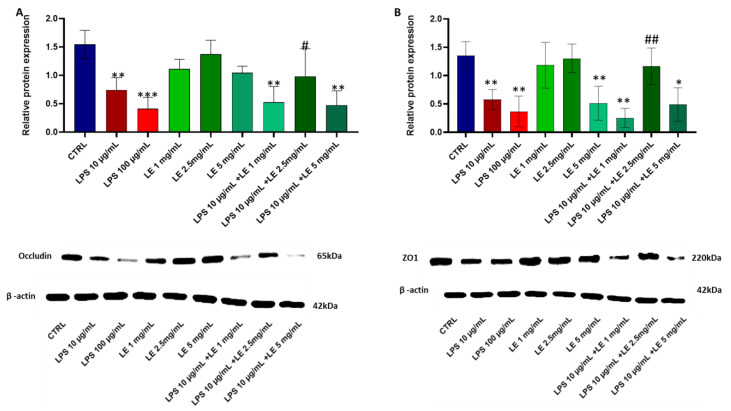
Effects on TJ protein expression in Caco-2 cell monolayers treated with LE, LPS, and co-incubation at different concentrations. (**A**) above panel: relative occludin expression normalized to beta-actin expression. Below panel: Western blotting images show occludin protein expression. (**B**) above panel: Relative ZO-1 expression normalized to beta-actin expression. Below panel: Western blotting images show ZO-1 protein expression. The results are expressed as the mean ± SD of a minimum of three independent experiments. * *p* < 0.05, ** *p* < 0.01, *** *p* < 0.001 vs. the control group, # *p* < 0.05, ## *p* < 0.01 compared to the LPS 10 μg/mL group.

Based on our initial experimental findings, we pinpointed the optimal concentration of LE at 2.5 mg/mL to counteract the effects of LPS at 10 μg/mL, as illustrated in Figure 2A,B. The administering of LE alone at doses of 1 mg/mL and 2.5 mg/mL did not induce significant alterations in occludin and ZO-1 expressions within the Caco-2 cell monolayer after a 48 h incubation period. On the contrary, a 5 mg/mL concentration of LE resulted in a relative protein expression reduction, probably due to a cytotoxic effect. Instead, when determining the LPS concentration, we deliberately selected a level known to provoke tight junction damage (Figure 2), while ensuring that the decline in cell viability remains under 25% (as depicted in Figure 1). This strategic decision was aimed at preserving cell viability within acceptable thresholds and sustaining the reliability of our experimental approach setup.

### 2.3. Effects of LE on TEER in LPS-Induced Intestinal Epithelial Barrier Dysfunction

**Figure 3:**
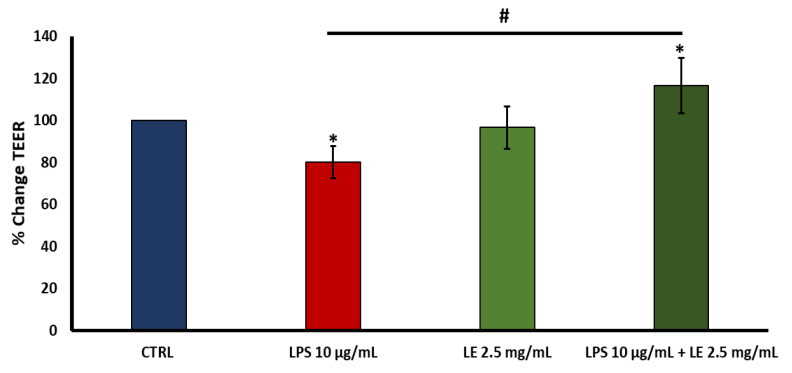
Effects of LE on trans-epithelial electrical resistance (TEER) in LPS-treated Caco-2 cell monolayers after 24 h incubation. The data were the mean ± SD of a minimum of three replications. The results are expressed as the mean ± SD of a minimum of three independent experiments with six replicates per experimental condition in each experiment. * *p* < 0.05 vs. the control group (CTRL), # *p* < 0.05 compared to the LPS group.

TEER testing was conducted to investigate the potential protective LE effects against the disruption of integrity induced by LPS in the Caco-2 cell monolayer. The control group served as the baseline reference. Following a 24 h incubation of Caco-2 cells with 10 μg/mL of LPS, a significant decrease in TEER of approximately 19.94% compared to the baseline (*p* < 0.05) was observed (Figure 3).

However, when Caco-2 cells were co-treated with LE, the treatment with a 2.5 mg/mL concentration significantly ameliorated the decrease in TEER induced by 10 μg/mL of LPS, exhibiting an improvement of approximately +16.61% compared to that of the control group (*p* < 0.05) (Figure 3). Instead, treatment of cells with LE at a concentration of 2.5 mg/mL did not determine any significant changes in TEER. This enhancement in TEER suggests that LE exerted a protective effect on the integrity of the intestinal epithelial barrier, mitigating the disruption caused by LPS.

### 2.4. The Impact of LEon the Expression of TJ Proteins during LPS-Induced Intestinal Epithelial Barrier Dysfunction

**Figure 4:**
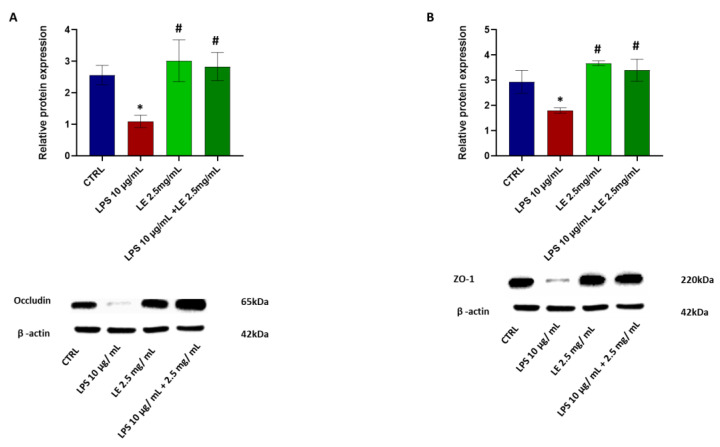
LE effect on TJ protein expression in Caco-2 cell monolayers treated with LPS. (**A**) above panel: Relative occludin expression normalized to beta-actin expression. Below panel: Western blotting images show occludin protein expression. (**B**) above panel: Relative ZO-1 expression normalized to beta-actin expression. Below panel: Western blotting images show ZO-1 protein expression. The results are expressed as the mean ± SD of a minimum of three independent experiments. * *p* < 0.05 vs. the control group, # *p* < 0.05 compared to the LPS group.

Occludin and ZO-1 expressions were assessed through Western blotting and qRT-PCR analyses to elucidate the potential positive impact of LE treatment on LPS-induced injury to the intestinal epithelial barrier. In the LPS-treated group, the protein levels of occludin (Figure 4A) and ZO-1 (Figure 4B) displayed a statistically significant (*p* < 0.05) decrease of approximately 33.08% and 38.85%, respectively, compared to that of the CTRL group. However, co-administration of LPS and LE resulted in a significant (*p* < 0.05) increase in the levels of these proteins, counteracting the damage induced by LPS, with improvements of approximately 86.03% and +88.95%, respectively vs. that of the LPS group.

**Figure 5:**
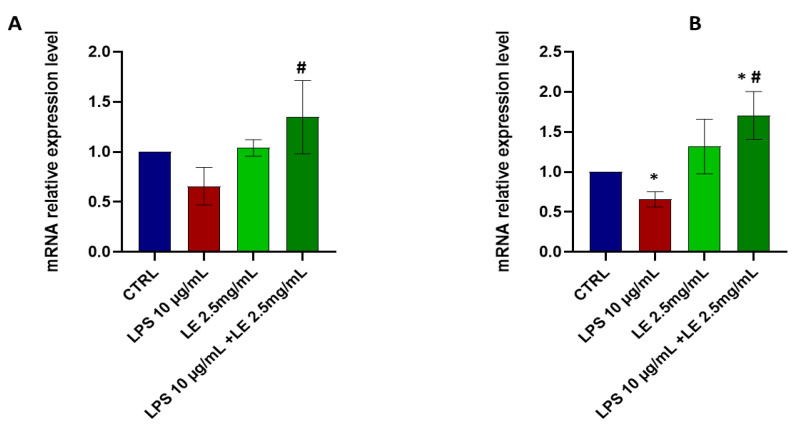
The mRNA expression levels of occludin (**A**); and ZO-1 (**B**). The results are expressed as the mean ± SD of a minimum of three independent experiments with three replicates per experimental condition in each experiment. * *p* < 0.05 vs. the control group, # *p* < 0.05 compared to the LPS group.

Similarly, in the LPS-treated group, the mRNA expression of ZO-1 (Figure 5B) exhibited a significant decrease (*p* < 0.05) compared to that of the CTRL group. Conversely, cells treated with LE alone or co-treated with LPS and LE did not show statistically significant (*p* < 0.05) changes in occludin and ZO-1 mRNA expressions compared to those of the CTRL group. It is noteworthy that both the LPS-alone group, and the group co-incubated with LE, exhibited statistically significant changes (*p* < 0.05) in the mRNA expression of occludin (Figure 5A) and ZO-1 (Figure 5B). Instead, treatment of cells with LE alone at a concentration of 2.5 mg/mL resulted in non-significant increases in the protein levels of occludin and ZO-1 vs. that of the LPS-alone condition and also vs. that of the CTRL group.

### 2.5. LE Treatment Effect on TLR-4 Modulation

**Figure 6:**
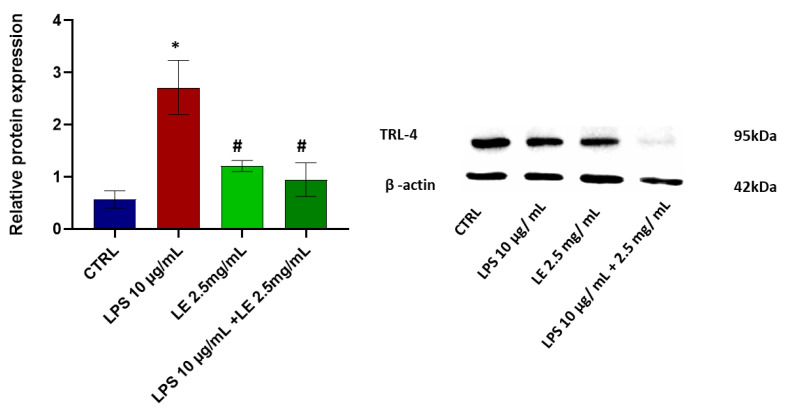
The effects of LE on the protein expression of TLR-4 in LPS-treated Caco-2 cell monolayers. **Left**: relative protein expression normalized to beta-actin expression. The results are expressed as the mean ± SD of a minimum of three independent experiments with three replicates per experimental condition in each experiment. * *p* < 0.05 vs. the control group, # *p* < 0.05 compared to the LPS group. **Right**: Western blotting images show TLR-4 protein expression.

The protein TLR-4 levels statistically and significantly (*p* < 0.05) increased with a percentage exceeding 100% compared to the CTRL group following a 24 h treatment with LPS. However, the LE co-incubation modulated the expression level of TLR-4, inducing a significant (*p* < 0.05) decrease compared to the LPS conditions alone. This decrease amounted to about 67.32% compared to that of the CTRL group and a substantial reduction of approximately 55.62% compared to the LPS-alone condition (Figure 6). Instead, treatment of cells with LE alone at a concentration of 2.5 mg/mL determined a slight and statistically insignificant increase vs. CTRL but a statistically significant decrease vs. the LPS-alone condition (Figure 6).

### 2.6. Effects of LE Treatment on LPS-Induced Inflammation

**Figure 7:**
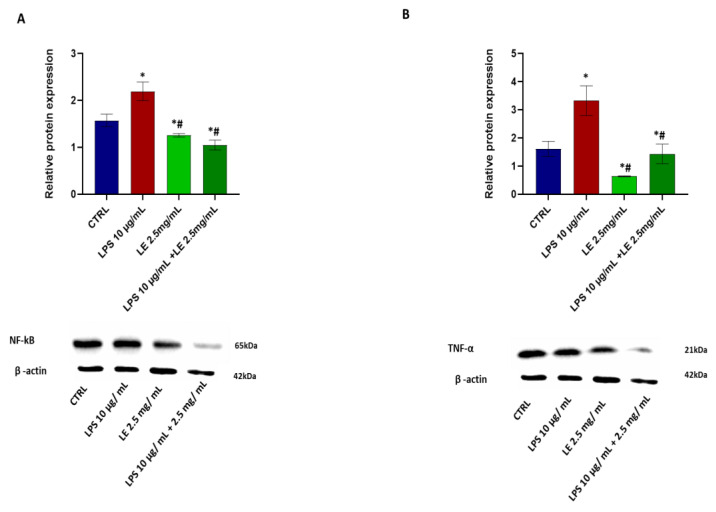
Effects of LE on LPS-induced inflammation. (**A**) above panel: relative TNF-α expression normalized to beta-actin expression. Below panel: Western blotting images show TNF-α protein expression. (**B**) above panel: Relative NF-κB expression normalized to beta-actin expression. Below panel: Western blotting images show NF-κB protein expression. The results are expressed as the mean ± SD of a minimum of three independent experiments with six replicates per experimental condition in each experiment. * *p* < 0.05 vs. the control group, # *p* < 0.05 compared to the LPS group.

**Figure 8:**
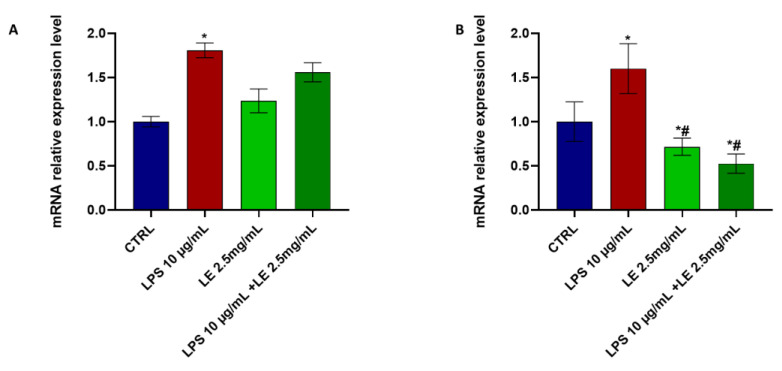
mRNA expression levels of IL-8 (**A**); and IL-1β (**B**). The results are expressed as the mean ± SD of a minimum of three independent experiments with six replicates per experimental condition in each experiment. * *p* < 0.05 vs. the control group, # *p* < 0.05 compared to the LPS group.

Treatment of Caco-2 cells with LPS alone led to a significant increase in the expression levels of NF-κB (Figure 7A) and TNF-α (Figure 7B) proteins, showing an elevation of approximately 25.57% and 109.8%, respectively, relative to the control. This same trend was observed in the mRNA levels of IL-8 (Figure 8A) and IL-1β (Figure 8B), which also increased by approximately 80.78% and 60%, respectively, relative to the CTRL.

However, co-incubation of Caco-2 cells with LPS and LE significantly restored the levels of NF-κB, TNF-α, and IL-1β (Figure 7 and Figure 8), indicating a mitigating effect on the inflammatory response induced by LPS. Treatment of cells with LE alone at a concentration of 2.5 mg/mL determined a statistically significant decrease vs. the LPS-alone condition and the CTRL in the expression levels of NF-κB (Figure 7A) and TNF-α (Figure 7B), and also determined a statistically significant decrease in mRNA level IL-1β (Figure 8B).

### 2.7. Effects of LE Treatment on the Protein Expression of AQP8

**Figure 9:**
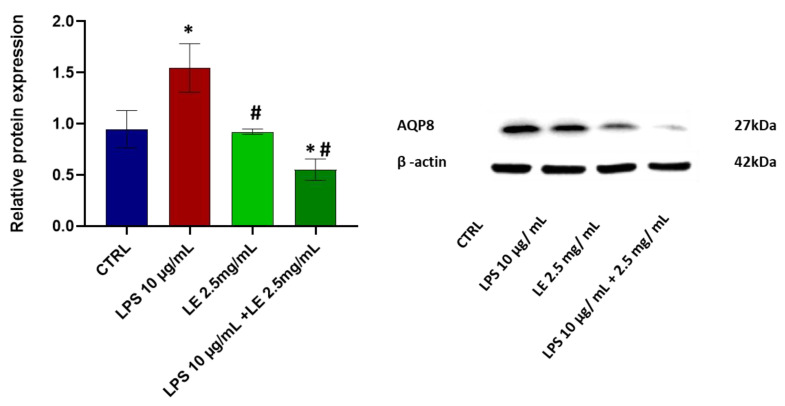
Effects of LE on: (**left**) relative protein expression of AQP8 normalized to β-actin expression. Error bars represent the standard deviation. * *p* < 0.05 compared to the control group (CTRL). # *p* < 0.05 compared to the LPS group; and (**right**) Western blotting images show AQP8 protein expression.

The protein level of AQP8 underwent a substantial and statistically significant (*p* < 0.05) increase of approximately 63.07% compared to the control condition after 24 h of LPS treatment. However, LE co-incubation modulated the expression of AQP8 protein, inducing a significant (*p* < 0.05) decrease compared to those of the control and LPS conditions alone. This decrease amounted to about 41.62% compared to the control and a notable reduction of approximately 64.20% compared to the LPS-alone condition (Figure 9). Treatment with LE alone at a concentration of 2.5 mg/mL determined a statistically significant decrease vs. LPS alone in the expression protein level of AQP8 (Figure 9).

### 2.8. LE Treatment Modulates TRPV1 and TRPA1 mRNA Expression in Caco-2 Cells

**Figure 10:**
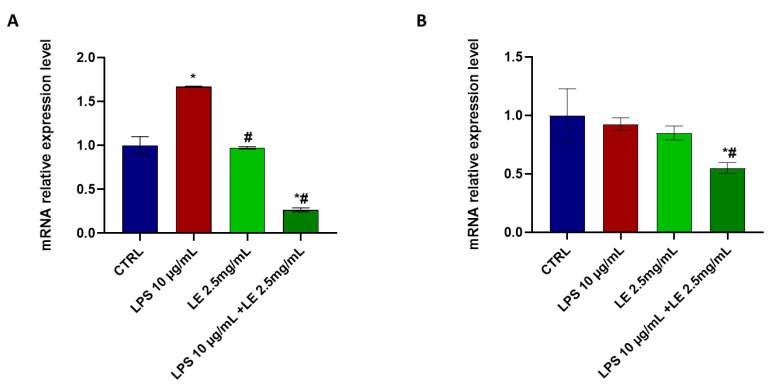
mRNA expression levels of *trpv1* (**A**); and *trpa1* (**B**). Error bars represent the standard deviation. * *p* < 0.05 compared to the control group (CTRL). # *p* < 0.05 compared to the LPS group.

LE treatment resulted in a downregulation of the expression of TRPV1 (Figure 10A) in Caco-2 cells. Specifically, exposure to LPS alone led to a significant 66.87% increase in the mRNA expression level of TRPV1 compared to the control condition (Figure 10A). However, the mRNA expression level of TRPA1 showed a non-significant increase under the same circumstances (Figure 10B).

In contrast, co-treatment with LE resulted in a significant downregulation of *trpv1* and *trpa1*, with reductions of approximately 73.35% and 44.7%, respectively, compared to that of the control. These findings highlight the significance of the differences in mRNA expression levels of *trpv1* and *trpa1* between the LPS-only treatment and LE co-treatment conditions, where both genes exhibit significant downregulation (Figure 10).

### 2.9. Ion Channel Currents in Caco-2 Cells

Whole-cell current recordings were conducted in Caco-2 cells under physiological conditions, revealing the presence of both outward and inward currents with similar intensities and a linear current-voltage (I/V) relationship. The cells exhibited a marked depolarization, with a membrane potential of 50.5 ± 4 mV, indicating the existence of depolarizing current components.

**Figure 11:**
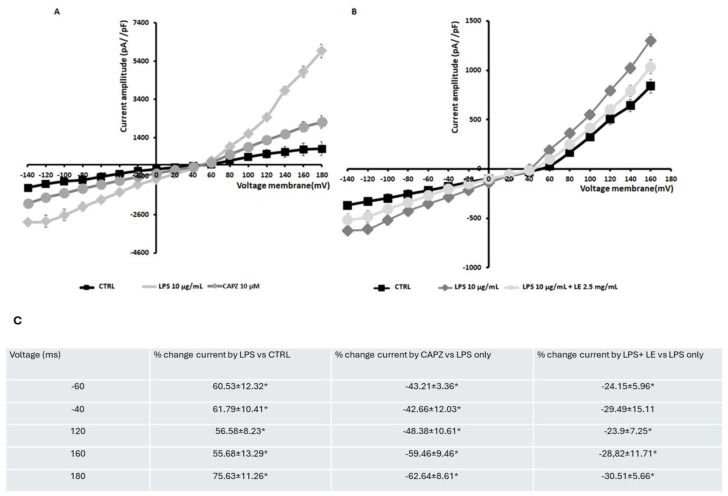
Current–voltage relationship of the whole-cell K^+^ current recorded in Caco-2 cells. (**A**) Characterization of the maximal activation effect of applying the LPS. The sigmoid curve I/V relationship of the K^+^ current in a single Caco-2 cell recorded in the control condition (CTRL) and in the presence of the specific LPS at 10 μg/mL at which currents were activated, especially at high voltages; these currents underwent an inhibition with capsazepine at a concentration of 10^−5^ M. (**B**) The effect of LE on currents activated by LPS. (**C**) Summary table of the % activation or blocking of physiological and high voltage currents and voltages recorded in whole cell mode using LPS or LPS + LE. Membrane currents were activated by 500 ms voltage steps between −140 to +180 mV, starting from an HP of −60 mV (Vm). Cells characterized by the same size were selected for patch-clamp experiments. Each point represented the mean ± SD of 5–8 experimental points. * *p* < 0.05.

Upon exposure to LPS, the cells displayed a substantial increase in outward current and augmented inward current, albeit with lesser intensity, resulting in a sigmoidal I/V relationship. These currents were significantly inhibited by the transient receptor potential vanilloid 1 (TRPV1) antagonist capsazepine (CAPZ) at a concentration of 10–5 M, suggesting the functional presence of TRPV1 channels (Figure 11A). CAPZ reduced the outward current at +180 mV (membrane potential) by approximately 70% compared to the LPS-induced currents, indicating the substantial contribution of TRPV1 to this current.

While an additional depolarizing current component cannot be ruled out, the cells’ membrane potentials, being close to the equilibrium potentials for chloride ions, suggest the existence of chloride channels in these cells.

When LE was administered at a concentration of 2.5 mg/mL in the presence of LPS at 10 µg/mL, it primarily activated currents at high voltages, with a notable inhibition of the outward currents observed (Figure 11B). Furthermore, LE caused a reduction in inward currents (Figure 11B). These data suggest that by inhibiting TRPV1 channels and other inward-rectifier potassium channels, such as Kir, LE likely mediates its effects against LPS in Caco-2 cells.

## 3. Discussion

The current study aimed to examine the impact of LE extract on the functionality of the intestinal barrier and its influence on ion channels like TRPV1, TRPA1, and AQP8, which are associated with pain relief and anti-inflammatory effects. Additionally, we investigated the effects of LPS and LE on TRPV1 currents in an in vitro model, utilizing whole-cell recording through the patch-clamp technique under physiological conditions.

As previous research indicates, LE exhibits noteworthy health benefits and bioactive properties [15]. In vitro studies have demonstrated its potential to protect against cytotoxic damage induced by cisplatin, irinotecan, and doxorubicin in various cell lines [15,16]. Its anti-inflammatory properties make it a promising candidate for managing inflammatory conditions.

To achieve our objectives, we employed a Caco-2 cell monolayer model treated with LPS alone or combined with LE. These cells, possessing brush borders, tight junctions (TJs), and other intestinal proteins similar to human intestinal epithelial cells upon differentiation, serve as a well-established model for studying small intestinal barrier function [17].

Moreover, LPS, a significant component of Gram-negative bacteria’s outer membrane, was utilized to induce intestinal epithelial barrier dysfunction. LPS is crucial in initiating inflammatory responses within the intestine [18]. Prolonged inflammatory stimulation can compromise the function of the intestinal TJ barrier, giving rise to pro-inflammatory cytokines and establishing a detrimental cycle. Consequently, LPS induces injury in Caco-2 cell monolayers, creating a model mirroring human intestinal epithelial barrier dysfunction.

Exposure of the Caco-2 cell monolayers to LPS at a concentration of 10 μg/mL resulted in a 25% reduction in cell viability and a 20% decrease in TEER [19]. This finding aligns with the existing literature indicating that even a lower LPS concentration of 10 μg/mL can inflict damage to the intestinal barrier [20,21]. Consequently, a 10 μg/mL concentration of LPS was selected to induce intestinal barrier damage in subsequent experiments.

As for LE, its liquid chromatography-mass spectrometry (LC-MS) analysis revealed a high content of flavonoids, including quercetin, which is known to reduce inflammation [22,23,24], mitigate intestinal barrier damage, and reduce paracellular permeability [25,26,27]. Flavonoids, as polyhydroxy compounds, interact with the structural proteins of the barrier. Specifically, their hydroxyl groups form hydrogen bonds with the -NH_2_ group of amino acids, creating a stable physical barrier that repels lipids, hindering the absorption of fatty acids. The complex formed by flavonoids, -OH, NH_2_, and protein exhibits minimal solubility in both aqueous and lipid-based fluids but readily dissolves in ethanol. Moreover, flavonoids interact with soluble food proteins, enhancing their availability and biological activity [28,29].

In our study, LE significantly countered the decrease in TEER values caused by LPS treatment, indicating its protective effect on the integrity of the intestinal epithelial barrier. Furthermore, LE positively influenced the integrity of Caco-2 cell monolayers by upregulating the expressions of ZO-1 and occludin while reducing pro-inflammatory cytokine levels (TNF-α, NF-κB, IL-8, and IL-1β). These cytokines, induced by LPS, can disrupt TJ proteins and lead to intestinal epithelial barrier dysfunction, as supported by the literature [30,31].

Evidence suggests that interferon-gamma (IFN-γ) and TNF-α can directly disrupt the function of ZO-1 and occludins [32,33]. The increased expression of these proteins in response to inflammatory challenges could be a direct outcome of LE’s positive and anti-inflammatory effects.

The primary goal of our current investigation was to examine AQP8, a member of the aquaporin protein family known to facilitate water transport across cellular membranes. AQP8 is expressed at both the apical and intracellular levels and shows cytoplasmic distribution in the epithelial cells of the colon. The existing literature emphasizes AQP8’s significant role in functional gastrointestinal disorders [34]. Research has revealed a connection between inflammatory states and increased AQP8 expression mediated by cytokines, NF-κB, and other mediators [35].

In contrast, documented evidence clarifies flavonoids’ role in modulating AQPs’ activity, facilitating water movement even in stressful and inflammatory conditions [35]. Given the immune responses and inflammation associated with irritable bowel syndrome (IBS), our hypothesis focuses on the LPS-activated NF-κB pathway in our cellular model, highlighting its pivotal role in regulating AQP8 expression in the context of IBS. The protective mechanism of LE involves inhibiting pro-inflammatory factors and suppressing the expression of the LPS receptor TRL-4, potentially leading to reduced NF-κB protein levels and, consequently, decreased AQP8.

Flavonoids interact with nuclear receptors and transcription factors, downregulating TRPA1 and TRPV1 genes. This demonstrates their anti-estrogenic activity and effectiveness in mitigating damage to the intestinal epithelial barrier. Additionally, flavonoids can modulate the aryl hydrocarbon receptor (AhR), a transcription factor forming an active nuclear heterodimer with the AhR nuclear translocator (Arnt) protein, activating gene expression [27]. Our findings support existing studies indicating a negative AhR–TRPV1 interaction, as our EL induces downregulation in the mRNA expression of TRPA1 and TRPV1, likely through the aforementioned mechanism.

Furthermore, flavonoids’ influence on G protein-coupled receptors (GPR), such as GPR30, likely plays a significant role in addressing intestinal disorders and could contribute to downregulating ion channel genes [36]. It is noteworthy that treating cells with LPS enhanced the functional and expression levels of the TRPV1 gene in qRT-PCR experiments. However, the LPS-induced currents were effectively inhibited by the selective TRPV1 antagonist CAPZ. These results support the pivotal role of TRPV1 channels in mediating ion channel currents, facilitating the entry of bivalent cations, including Ca^2+^ ions, which can trigger the inflammatory cascade. EL treatment reduces the influx of Ca^2+^ ions, preventing the cascade of calcium-dependent reactions and contributing to the anti-inflammatory action of EL by downregulating the TRPV1 gene.

In conclusion, the present work, showing the protective effects of LE on a cellular model such as Caco-2 cells, represents an essential in vitro step. Still, as expected, the evaluation and confirmation of the effects of LE in an animal model are mandatory because they alone do not provide conclusive results. However, these investigations could be particularly useful as a starting point for future applications in human clinical studies, particularly regarding gastrointestinal disorders.

## 4. Materials and Methods

### 4.1. Chemicals and Reagents

LE was obtained from Lens culinaris supplied by Terre di Altamura Srl Bari, Italy. Based on these results and previous studies on various flavonoids in monolayer cell models [15,16], 2.5 mg/mL of LE was selected for the subsequent experiments. The concentration of LE at 2.5 mg/mL was chosen due to its cytoprotective activity in different cells observed in previous in vitro experiments [15,16].

Cell culture reagents and other chemicals were provided by Sigma-Aldrich, Milan, Italy). For the patch-clamp experiments, the pipette solution contained 132 mM KCl (P3911), 1 mM ethylene glycol-bis (β-aminoethylether)-*N*,*N*,*N*′,*N*′-tetraacetic acid EGTA (E3889, Sigma-Aldrich, Milan, Italy), 10 mM NaCl (S9888, Sigma-Aldrich, Milan, Italy), 2 mM MgCl_2_ (M8266, Sigma-Aldrich, Milan, Italy), 10 mM HEPES (H3375, Sigma-Aldrich, Milan, Italy), 1 mM Na_2_ATP (A26209, Sigma-Aldrich, Milan, Italy), and 0.3 mM Na_2_GDP (51060, Sigma-Aldrich, Milan, Italy) (pH = 7.2). The bath solution contained 142 mM NaCl (S9888, Sigma-Aldrich, Milan, Italy), 2.8 mM KCl (P3911, Sigma-Aldrich, Milan, Italy), 1 mM CaCl_2_ (C8106), 1 mM MgCl_2_ (M8266), 11 mM glucose (D9434), and 10 mM HEPES (H3375, Sigma-Aldrich, Milan, Italy) (pH = 7.4) [37,38,39]. In the whole-cell experiments, CaCl_2_ was added to the pipette solutions to give a free Ca^2+^ ion concentration of 1.6 × 10^−6^ M. The free Ca^2+^ ion concentration in the pipette was calculated using the MaxChelator software (maxchelator.stanford.edu; Stanford University, Stanford, CA, USA). TRPV1 modulators, capsaicin (CAPS, M2028, Sigma-Aldrich, Milan, Italy)/capsazepine (CAPZ, C191, Sigma-Aldrich, Milan, Italy).

Primary antibodies AQP8 (Cat# AF5222, Abcam, Cambridge, UK), NF-κB8 (Cat# 10745-1-AP, Abcam), TRL-4 (Cat# ab217274, Abcam), β-actin (Cat# 4970S, Cell Signaling Technology, Danvers, MA, USA), ZO-1 (Cat# 8193T, Cell Signaling Technology), occludin (Cat# 91131S, Cell Signaling Technology).

TaqMan hydrolysis primer and probe gene expression assays were obtained from Life Technologies (Carlsbad, CA, USA).

### 4.2. Cell Line and Culture Conditions

A human colon adenocarcinoma-derived Caco-2 cell line was obtained from the Interlab Cell Line Collection (IST, Genoa, Italy) and routinely cultured in RPMI-1640, 10% fetal bovine serum (FBS), 2 mM glutamine, 100 U/mL penicillin, 100 μg/mL streptomycin, in a monolayer culture at 37 °C in 5% CO_2_. All reagents were from Sigma Aldrich (Milan, Italy).

The experimental design of this study consisted of exposure of Caco-2 cells to LPS alone (5–100 µg/mL) or co-administration with LE (2.5 mg/mL) for 24 h. After 24 h of treatment, cells were immediately harvested and processed for protein or mRNA extraction on the same day. Each treatment included its control (CTRL) (untreated cells). Each experimental condition was conducted in triplicate.

### 4.3. Viability Assay on Caco-2

Cell viability was assessed using the 3-(4,5–dimethylthiazol-2-yl)-2,5–diphenyltetrazolium bromide (MTT) test. This assay is based on the principle that mitochondrial dehydrogenases primarily reduce MTT tetrazolium salt. Following a 24 h exposure or after specific incubation periods in the culture medium, an MTT stock solution (5 mg/mL in medium) was added to each dish at a volume equivalent to one-tenth of the original culture volume. The cells were then incubated for 2 h at 37 °C in a humidified CO_2_ environment. Subsequently, the culture medium was replaced with acidic isopropanol (0.1 N HCl in absolute isopropanol), used to solubilize the formazan crystals formed due to the MTT reduction. Formazan formation was quantified using spectrophotometry, measuring the absorbance at 570 nm. The amount of formazan produced is proportional to the number of viable cells in the culture, allowing for the evaluation of cell viability under different experimental conditions.

### 4.4. Measurement of Trans-Epithelial Electrical Resistance (TEER)

Trans-epithelial electrical resistance (TEER) is a valuable measure used to assess the fusion and integrity of a cellular monolayer. For this study, Caco-2 cells were seeded at a density of 1.5 × 10^4^ cells per well in transwell plates with polyester membranes (0.33 cm^2^, 0.4 μm pore size) obtained from Corning, MA, USA. The cells were closely monitored until the TEER values reached a level greater than 300 Ω·cm^2^, indicating the establishment of a tight and intact monolayer; this value was reached after the fifteenth day of sowing. This TEER value signifies the successful formation of cell-to-cell junctions, which are crucial for maintaining the integrity of the epithelial barrier.

Subsequently, the cells were exposed to different treatments. Specifically, they were treated with LE alone at 2.5 mg/mL, lipopolysaccharide (LPS) alone at a concentration of 10 µg/mL, or co-administered with LE at 2.5 mg/mL. These treatments were applied for 24 h. After the treatment period, TEER was measured using a Millicell^®^ ERS-2 voltmeter from Merck Millipore, Darmstadt, Germany. Before the measurement, the cells were washed twice with phosphate-buffered saline (PBS) to ensure the accuracy of the TEER readings. TEER values were then calculated using the formula: TEER (Ω·cm^2^) = (Cell resistance − Cell-free resistance) × 0.33 cm^2^.

### 4.5. Western Blotting Analysis

To obtain protein extracts, pellets from both control and treated cells were treated with a total lysis buffer. The total lysis buffer used in this study was Pierce RIPA buffer, manufactured by Thermo Scientific (Rockford, IL, USA). Additionally, protease and phosphatase inhibitors (Thermo Scientific, Rockford, IL, USA) were supplemented to the lysis buffer to prevent the degradation of proteins and dephosphorylation, respectively. Following homogenization and centrifugation at 14,000 rpm for 15 min at 4 °C, the protein concentration was determined using a standard Bradford assay (Bio-Rad, Milan, Italy). Subsequently, 30 µg aliquots of total protein extracts from each sample were denatured in a 5× Laemmli sample buffer and loaded into pre-cast polyacrylamide gels (4–12%) from Bio-Rad (Milan, Italy) for Western blot analysis. To detect all primary antibodies, a dilution at 1:1000 except for anti-β-actin (dilution at 1:7000) was used (A2066, Sigma Aldrich, Milan, Italy). The membranes were incubated overnight with these primary antibodies and then exposed to a horseradish peroxidase-conjugated rabbit secondary antibody at 1/5000. Chemiluminescence (Clarity Western ECL substrate, Bio-Rad, Milan, Italy) was employed to detect the proteins, and the signals were analyzed using the ChemiDoc System (Model No. Universal Hood III) and Image Lab version 6.1 software from Bio-Rad Laboratories Inc. (Hercules, CA, USA). To normalize each band’s densitometric values (OD units), β-actin expression was utilized as a reference.

### 4.6. Quantitative Real-Time Polymerase Chain Reaction (qRT-PCR) Analysis

Total RNA was isolated and purified from the Caco-2 cells using an RNA extraction kit (Cat. number 74104, QIAGEN, Hilden, Germany) following the manufacturer’s instructions and quantified using a spectrophotometer (ND-1000 Nano-Drop, Thermo Fisher Scientific Inc., Waltham, MA, USA). Subsequently, the extracted RNA was subjected to reverse transcription using a First Strand cDNA Synthesis Kit provided by Service bio, Wuhan, China. Real-time PCR was performed in triplicate using the Applied Biosystems Real-time PCR 7500 Fast system (United States). The mRNA expression of the genes was normalized to the best housekeeping gene, β-actin (Actb). TaqMan hydrolysis primer and probe gene expression assays were obtained from Life Technologies with the following assay TRPA1 ID: qHsaCIP0027750; TRPV1 ID: qHsaCIP0033268; B-actin: ID: qHsaCEP0036280; IL-8 ID: qHsaCEP0053894; IL-1β ID: qHsaCIP0033362.

### 4.7. Patch-Clamp Experiments

The whole-cell patch-clamp experiments for membrane currents recorded were performed in asymmetrical K^+^ ion concentration in physiological conditions using pipettes with a resistance of 3–5 MΩ. Drug actions on the K^+^ ion currents recorded during instantaneous I/V relationships were investigated by applying a depolarization protocol in response to voltage pulses from −100 mV to +200 mV (Vm) in 20 mV steps. Currents were expressed as densities (pA/pF) to control for cell size/capacitance differences. All the experiments were performed in conditions like those described by Maqoud et al. [40]. The solution applications have been performed as described previously in [41].

### 4.8. Statistical Analysis

Data were analyzed by one-way ANOVA analysis of variance and Dunnett’s multiple comparison tests for treated vs. untreated control cells. All data are expressed as mean and SD of at least three independent experiments, with six replicates per experimental condition in each experiment. Differences were considered significant at *p* < 0.05. A specific software package was used for the statistical analysis (The R Project for Statistical Computing version 4.1.3) [42].

## 5. Conclusions

Our research revealed that LE successfully maintained the integrity of TJs within Caco-2 monolayers, resulting in the mitigation of intestinal epithelial barrier dysfunction induced by LPS. This positive outcome was evident in reducing pro-inflammatory marker release and inhibiting specific signaling pathways. Notably, a downregulation of receptors like TRL-4, TRPA1, TRPV1, and AQP8 occurred. These findings highlight the functional attributes of natural compounds such as LE and provide a robust basis for considering this substance as a potential supplement in functional foods to enhance intestinal health and counteract dysfunction in particular gastrointestinal diseases.

## Data Availability

The datasets used and/or analyzed during the current study are available from the corresponding author upon reasonable request.
